# Emerging Role of Exosomes in Liquid Biopsy for Monitoring Prostate Cancer Invasion and Metastasis

**DOI:** 10.3389/fcell.2021.679527

**Published:** 2021-05-04

**Authors:** Zhengfan Gao, Bairen Pang, Jing Li, Na Gao, Tianli Fan, Yong Li

**Affiliations:** ^1^Department of Pharmacology, School of Basic Medicine, Zhengzhou University, Zhengzhou, China; ^2^Faculty of Medicine, St George and Sutherland Clinical School, St George Hospital, UNSW Sydney, Kensington, NSW, Australia

**Keywords:** prostate cancer, exosome, invasion and metastasis, biomarker, liquid biopsy

## Abstract

Prostate cancer (PCa) is the most common solid tumor in men. While patients with local PCa have better prognostic survival, patients with metastatic PCa have relatively high mortality rates. Existing diagnostic methods for PCa rely on tissue biopsy and blood prostate-specific antigen (PSA) detection; however, the PSA test does not detect aggressive PCa. Liquid biopsy is a promising technique to overcome tumor heterogeneity in diagnosis, provide more comprehensive information, and track tumor progression over time, allowing for the development of treatment options at all stages of PCa. Exosomes containing proteins and nucleic acids are potential sources of tumor biomarkers. Accumulating evidence indicates that exosomes play important roles in cell communication and tumor progression and are suitable for monitoring PCa progression and metastasis. In this review, we summarize recent advances in the use of exosomal proteins and miRNAs as biomarkers for monitoring PCa invasion and metastasis and discuss their feasibility in clinical diagnosis.

## Introduction

Prostate cancer (PCa) is one of the most common cancers among men in Western countries (Culp et al., 2020). The incidence of PCa in China has increased in recent years (Chen et al., 2016). Although patients diagnosed with localized PCa have a relatively high 5-year survival rate in most cases, metastatic PCas remain the leading cause of cancer-related deaths in men in Western countries (Fitzmaurice et al., 2019). According to the United States Surveillance, Epidemiology, and End Results (SEER) database, the 5-year survival rate of metastatic PCa is only 30.5%, while the 5-year survival rate of local PCa is nearly 100%. Therefore, distant metastasis in PCa patients is believed to be one of the most common causes of increased mortality.

PCa development has distinct stages. According to the guidelines of the European Society of Urology (EAU) in 2020, TNM grading method is recommended to stage PCa (Mottet et al., 2021). T (tumor): Whether the tumor cells are confined to the prostate tissue and whether they spread to surrounding tissues or organs. N (nodes): Whether there is lymph node metastasis. M (metastasis): Whether to transfer to distant tissues or organs. Early localized PCa can achieve good therapeutic effects through radical surgery or radical radiotherapy. However, PCa is prone to bone metastasis. In the late stage, PCa may develop into castrate-resistant prostate cancer (CRPC), which is very hard for the current treatments Therefore, the early diagnostic of PCa and the monitoring of cancer development are key steps in the radical cure of PCa.

Current diagnostic methods mainly rely on tissue biopsy and blood prostate-specific antigen (PSA) test. However, these two detection methods are not sensitive enough to detect early stage PCa and some aggressive tumors. Moreover, most of the abnormal PSA values are false positive results, which can be caused by benign prostatic hyperplasia (BPH), prostatitis or cystitis, and the normal value of PSA cannot exclude PCa (Hoffman, 2011). Therefore, there is an urgent need of new biomarkers for the diagnosis and prognosis of PCa. Because PCa is a heterogeneous and multifocal disease, multiple biomarkers are needed for its clinical diagnosis. Therefore, researchers are now turning to minimally invasive liquid biopsies. Liquid biopsy refers to the analysis of blood, urine, or other body fluids to obtain clinical or biologically relevant information about malignant tumors, which is better than the information obtained from traditional tumor biopsies (Merker et al., 2018). Liquid biopsies analyze body fluids using different methods to characterize different components, including circulating tumor cells (CTCs), cell-free tumor DNAs (ctDNAs), and extracellular vesicles (EVs). However, a liquid biopsy has advantages over tissue biopsy in reducing invasive injury and pain. Due to the heterogeneity of tumors, tissue biopsy may take several core biopsies, whereas a liquid biopsy is easy to collect (e.g., drawing blood).

During cancer development, signal transmission between cells plays a vital role in tumor formation, progression, and metastasis (Archer et al., 2020). Exosomes are one of subtypes of EVs that are 30–120 nm in diameter and are secreted by various types of cells under physiological and pathological conditions. During cancer progression and metastasis, exosomes are considered as a common central participant between cells (Bastos et al., 2018). The complex signaling pathway network between exosome-mediated cancer cells and the tumor microenvironment (TME) is considered as a key factor in the progression of cancer at all stages (Gulei et al., 2018). Studies have shown that exosomes play an important role in tumor immunoregulation, microenvironmental reorganization, angiogenesis, invasion, metastasis, and survival (Gulei et al., 2018). Recently, exosomes have become a promising tool for PCa diagnosis. When the number of exosomes increases or they enter fluid circulation, exosomes have the ability to induce drug resistance, angiogenesis, and metastasis (Saber et al., 2020). Researchers are increasingly interested in using liquid biopsies to diagnose early stage PCa or monitor PCa progression. From a clinical perspective, liquid biopsy can be used to predict the prognosis and the effect of PCa treatment or monitor the progression and metastasis of the disease. From a biological point of view, liquid biopsy, as a more accurate method, can reflect all the molecular characteristics of metastatic tumors, thus revealing the mechanism of drug resistance and paving the way for the development of new therapies.

In this review, we discuss the new developments in the field of liquid biopsy of PCa and focus on the application of exosomes as biomarkers for monitoring PCa invasion and metastasis.

## Exosomes in PCa

### Biogenesis and Function of Exosomes

**FIGURE 1:**
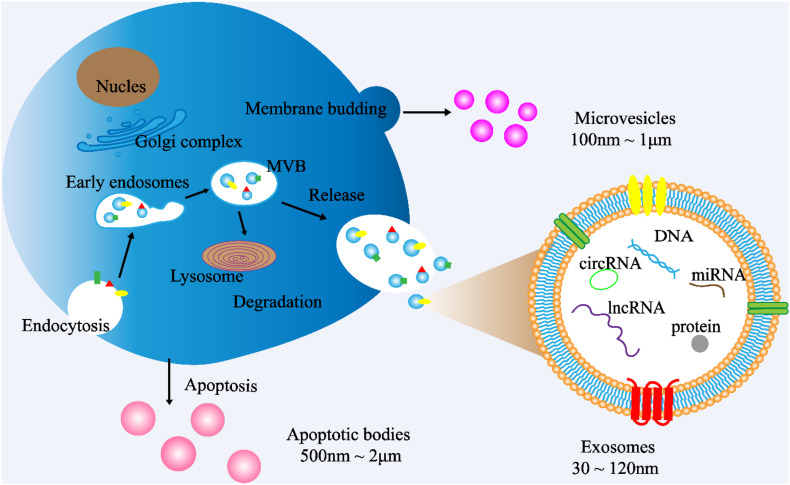
Exosome biogenesis. (1) Exosome: The cytoplasmic membrane endocytosis to form vesicles, which are then fused with early endosomes. Then, the early endosomes invade to form multivesicular bodies (MVBs). Finally, MVBs are fused with cell membranes and released outside the cell to become exosomes. Diameter: 30∼120 nm. (2) Microvesicles: Budding directly from the cell membrane. Diameter: 100 nm∼1 μm (3) Apoptotic bodies: Released when cells suffer from programmed death or late apoptosis. Diameter: 500 nm∼2 μm.

Cells communicate with each other by releasing different types of EVs, such as exosomes. EVs are small double membrane structures released by normal and abnormal cells, and divided into three main types according to the size of vesicles. The diameters of exosomes, microvesicles and apoptotic bodies are 30–120 nm, 100 nm to 1 μm, 500 nm to 2 μm, respectively. According to the International Society of Extracellular Vesicles (ISEV), the term “extracellular vesicles” is the appropriate terminology for heterogeneous populations of vesicles isolated from cell culture supernatants or physiological fluids (Witwer et al., 2013). Throughout this review, exosomes will be referred to as EVs. Exosomes are cup-shaped double-membrane nanovesicles that enter the local microenvironment and circulatory system. Exosomes are intraluminal vesicles derived from multivesicular bodies (MVBs) through the endosome maturation process, in which some vesicles are fused with lysosomes to degrade the substances contained in them, and other vesicles are fused with the plasma membrane, releasing the exosomes into the extracellular matrix (ECM) (Figure 1; Riches et al., 2014; Wei et al., 2017).

Different methods are used for isolating exosomes, including traditional ultracentrifugation and density gradient centrifugation, as well as ultrafiltration, immunoaffinity capture, PEG precipitation, and size exclusion chromatography (SEC) developed later (Yang et al., 2019). Each of these methods has advantages and disadvantages. At present, ultracentrifugation is a gold standard for the isolation of exosomes. The identification of exosomes is mainly based on three methods: transmission electron microscope (TEM), Nanosight particle size analysis (NTA), and protein marker analysis. The TEM resolution is 0.1–0.2 nm, which is suitable for the observation of the structure of the exosome double membrane. It can be used to observe whether there is an exosome-like structure in the sample (usually a saucer type or a hemispherical depression on one side). The size of exosomes can also be measured by TEM. NTA uses a laser light source to illuminate the nanoparticle suspension, and the Brownian motion of the particles with scattered light is clearly observed. NTA is often used to directly observe each particle at the same time, automatically track and calculate the particle size. Furthermore, there are specific marker molecules on the surface of exosomes, such as CD9, CD63, and CD81, which are identified by Western blot or Flow cytometry (Thery et al., 2018).

During cell changes, signal transmission between cells is essential for the cells to adapt to internal and external changes, such as embryonic development, stress response to injury, maintenance of homeostasis, and other functions (Pitt et al., 2016). Cell-to-cell communications involve the transmission of various signals through body fluids and circulation, with different mechanisms of direct contact or long-range interaction. Exosomes are present in body fluids, including the plasma, cerebrospinal fluid, and urine. As a material “transport carrier” in the circulated body fluids, exosomes play an important role in a variety of physiological and pathological processes due to their ability to carry a variety of proteins, nucleic acids, and lipids, transporting the contents to surrounding cells for inter-cell communication (Zhang and Grizzle, 2014). Tumor-derived exosomes participate in the formation and progression of different cancer processes, including tumor TME remodeling, angiogenesis, invasion, metastasis, and drug resistance (Mashouri et al., 2019).

As transport carriers, exosomes carry different signaling molecules in autocrine, paracrine, and endocrine modes. Exosomes mediate cell-to-cell signal transduction through the following pathways: (1) transfer biologically active molecules in exosomes to activate or inhibit signaling pathways in target cells; (2) shuttle receptors between donor and recipient cells to change cell activity; (3) transfer full-function proteins to perform specific functions in target cells; (4) provide new genetic information to the recipient cells to obtain new phenotypes (Saber et al., 2020).

### Exosome Functions in PCa

With the further study of nucleic acids, proteins, and lipids carried in exosomes, researchers have discovered that biologically active molecules in exosomes regulate the development of cancer in many ways through cell-cell interactions. Exosomes are key biomarkers for the early diagnosis of PCa, personalized treatment, and prognosis of patients (Vlaeminck-Guillem, 2018). Exosomes in blood and urine of PCa patients were reported to contain unique PCa-specific components, which are the source of biomarkers for PCa metastasis (Tavoosidana et al., 2011; Overbye et al., 2015). Hessvik et al. (2013) identified 36 exosomal miRNAs and proteins as candidate biomarkers for PCa in clinical studies. Huang et al. (2013, 2015) found that miR-1290 and miR-375 are potential prognostic biomarkers for CRPC.

A large amount of evidence has shown that exosomes play an important role in the occurrence, development, angiogenesis, metastasis, and tumor immune escape of PCa. For example, in the case of PCa, sphingomyelin and CD147 are transferred to endothelial cells through exosomes to promote vascularization (Naito et al., 2017). Newly formed blood vessels promote tumor growth by transporting tumor-derived secreted factors (TDSFs) and CTCs into secondary tissues (Guo et al., 2019). The exosomes secreted by PCa cells cause bone metastasis of PCa through the fusion and differentiation of osteoclasts (Hashimoto et al., 2018). Exosomes in the blood and urine of PCa patients contain unique PCa-specific bioactive molecules, which are potential biomarkers for PCa diagnosis or monitoring cancer metastasis. For example, exosomal miR-26a derived from PCa cells significantly changed the expression of epithelial–mesenchymal transition (EMT)-related factors and inhibited the metastasis and tumor growth of PCa (Wang et al., 2019). Exosomal integrin αvβ3 can also increase PCa aggressiveness (Krishn et al., 2019). These biologically active molecules in exosomes are promising key biomarkers for PCa diagnosis, metastasis detection, individualized treatment, and patient prognosis.

## Invasion and Metastasis of PCa

The main causes of death in cancer patients are invasion and metastasis. To survive in other organs, cancer cells must take various measures to leave the original site of the tumor to survive and grow in distant locations (Chaffer and Weinberg, 2011). The process of tumor metastasis includes detachment from the primary site of the tumor, entering the surrounding microenvironment, entering the circulation or lymphatic system, adhering to the endothelial cell wall and migrating out of the blood vessels, distant invasion, angiogenesis, and the formation of new metastatic foci. Accumulating evidence suggests that exosomes have a potential role in altering the TME and promoting aggressive tumor behavior (Saber et al., 2020). Exosomes released from the TME regulate proliferation, reduce apoptosis, promote angiogenesis, and regulate immune escape, thus promoting the invasion and metastasis of PCa (Lorenc et al., 2020). Tumor invasion and metastasis are dynamic and complex processes that are mainly related to the following factors.

### Extracellular Matrix Degradation

Cancer cell invasion is an early step in the metastatic cascade. Cancer-derived exosomes are directly involved in this process by remodeling the ECM and improving the ability of cancer cells to migrate and invade (Baghban et al., 2020). Cancer-derived exosomes bind to ECM components through adhesion receptors and release proteases, including matrix metalloproteinases (MMPs) and cathepsins, which degrade collagen, laminin, and fibronectin. Exosome and MMP secretion are necessary during the invasion and metastasis process, which can remodel and enzymatically hydrolyze the ECM, allowing cancer cells to invade the tissue barrier and metastasize (Brown and Murray, 2015; Syn et al., 2016). Metastatic PCa is determined by the expression of a rare isoform A of the molecular motor myosin IC, which binds to MMP-containing exosomes and stimulates exosome secretion, promoting the invasion of PCa cells through the ECM barrier (Maly et al., 2017). After ECM remodeling, it promotes the release of cytokines and growth factors, which affect other cells in the microenvironment (such as fibroblasts) to promote the migration and invasion of cancer cells (Colotta et al., 2009). For example, transforming growth factor (TGF-β) in tumor-derived exosomes was found to cause a signaling cascade in fibroblasts, inducing the differentiation of cancer-associated fibroblasts (CAFs) (Webber et al., 2010). In PCa, exosomes induce fibroblasts to differentiate into myofibroblasts in a TGF-β1-dependent manner, promoting tumor growth (Webber et al., 2015). Therefore, it is believed that exosomes can be used as chemokines to promote the migration of cancer cells.

### Angiogenesis

Cancer exosomes promote tumor heterogeneity and plasticity, vascular remodeling, tumor-niche co-evolution, immunomodulation, and the establishment of a pre-metastatic environment by regulating all aspects of cell function, all of which are important for the metastasis process (Wang, 2019). Tumor angiogenesis is synchronized with tumor growth. The rich vascular network can provide sufficient oxygen, necessary nutrients, and tumor growth factors for cancer cell growth and provide a perfect pathway for cancer cell metastasis. PCa-derived exosomes, as carriers of many lipids, proteins, and RNAs, have been reported to affect proliferation and angiogenesis (Vlaeminck-Guillem, 2018). It was found that the vesicle structure of exosomes could be used as a carrier of TGF-β, which induces the transformation of fibroblasts into myofibroblasts and promotes angiogenesis by activating the TGF-β/Smad3 signaling pathway or independent Smad signaling pathway (Muppala et al., 2017). Some researchers have found that TGF-β1 carried by exosomes induces a highly invasive myofibroblast phenotype and has high angiogenic activity (Webber et al., 2015; Pan et al., 2017).

Hypoxia is a common feature in many cancers. Hypoxia and angiogenesis often occur simultaneously. A recent report showed that the secretion of exosomes was enhanced under hypoxia, and these exosomes enhanced angiogenesis (Mashouri et al., 2019). In PCa, the process of angiogenesis is supported by the transformation of sphingomyelin and CD147 to endothelial cells through exosomes (Naito et al., 2017). The newly formed blood vessels were found to promote tumor growth by TDSFs and CTCs to secondary tissues, resulting in vascular leakage (Guo et al., 2019). Exosomes derived from cancer cells re-regulate the growth factors and cytokines secreted by endothelial cells and activate the signaling pathway, resulting in the migration of perivascular cells and the formation of new blood vessels (Kucharzewska et al., 2013). Cancer cells use exosomes to stimulate angiogenesis and promote disease invasion and metastasis (Mashouri et al., 2019).

### Metabolic Changes of Tumor Cells

Changes in cell metabolism are signs of cancer progression. According to the Warburg effect, cancer cells have a high tendency for glycolysis and the production of lactic acid, which reduced the pH of the TME, resulting in the progression and invasion of cancer (Warburg, 1956). In the TME, metabolic regulation between cancer cells and stromal cells plays an important role in the survival and growth of cancer cells. Stromal cells include CAFs, TAMs, bone marrow-derived cells (BMDCs), and tumor endothelial cells (TECs) (Tomasetti et al., 2017). The most common situation is that stromal cells support the growth of cancer by exosome exchange during the process of adapting to glycolysis. These exosomes provide metabolic intermediates for cancer cells, such as lactic acid, pyruvic acid, ketones, and glutamine, which can be used by cancer cells for the biosynthesis of large molecules (Tomasetti et al., 2017). Cancer-derived exosomes have been reported to induce the Warburg effect, improve the efficiency of glycolysis, and produce lactic acid in stromal cells, leading to cancer progression and invasion (Vander Heiden et al., 2009). Fibroblasts account for one third of the stromal cells and are key participants in cancer development (Martinez-Outschoorn et al., 2014). Studies have shown that exosomes secreted by CAFs increase the ability of PCa cells to proliferate and survive in hypoxic and low-nutrient environments by inhibiting mitochondrial oxidative phosphorylation, increasing anaerobic glycolysis, and promoting the progression of PCa (Vander Heiden et al., 2009; Webber et al., 2015).

### Immune Escape

**FIGURE 2:**
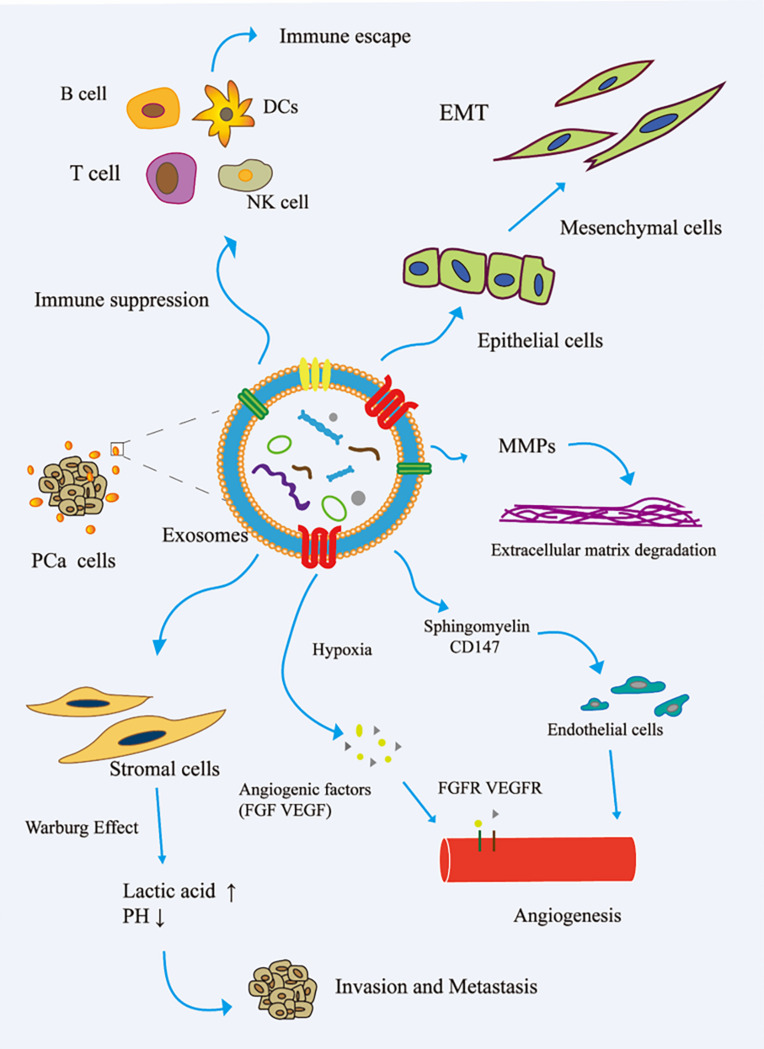
Role of exosomes in the invasion and metastasis of PCa. (1) The exosomes secreted by PCa bind to the components of ECM through adhesion receptors and released proteases, which makes the ECM reshape and be hydrolyzed by enzymes. In addition, in PCa, exosomes promote epithelial cells to mesenchymal cells. This allows cancer cells to invade the tissue barrier and metastasize. (2) In PCa, exosomes promote angiogenesis by transferring sphingomyelin and CD147 to endothelial cells; in the case of hypoxia, exosomes promote angiogenesis by promoting the secretion of angiogenic factors. (3) PCa cell-derived exosomes induce the Warburg effect, increase glycolysis rate, and produce lactic acid in stromal cells, leading to cancer progression and invasion. (4) In the tumor microenvironment, cancer cells remodel B cells, T cells, DCs, and NK cells via exosomes to promote tumor progression. EMT, epithelial–mesenchymal transition; FGF, fibroblast growth factor; FGFR, fibroblast growth factor receptor; MMP, matrix metalloproteinase; VEGF, vascular endothelial grown factor; VEGFR, vascular endothelial growth factor receptor.

The immune system is one of the main obstacles to tumor progression. The main mechanism of tumor cell immune escape is the change in the antigen presentation mechanism, which involves the downregulation or non-expression of the major histocompatibility complex class I molecule (MHCI) on the surface of the tumor cell membrane. Here, tumor cells inhibit the differentiation of myeloid progenitor cells into mature antigen-presenting cells. Therefore, the complex communication between immune cells and tumor cells is essential for the occurrence, development, and metastasis of cancer. The tumor regulation of immune cells includes the precise upregulation of the expression of genes and proteins, and then escapes the recognition and killing of immune cells (Eichmüller et al., 2017). In the TME, cancer cells reshape cytotoxic T lymphocytes (CTLs) and natural killer cells (NK) to promote tumor progression (Psaila and Lyden, 2009; Grivennikov et al., 2010). Cancer-related exosomes were found to interfere with the development of CD14^+^ monocytes into mature dendritic cells (DCs) (Gabrilovich and Nagaraj, 2009). More interestingly, activated BMDSCs interact with immune cells through exosomal HSP72, TLR-2, and MyD88, reducing the cytotoxic effects of NK cells and CD4^+^/CD8^+^ lymphocytes (Zhang and Grizzle, 2011). In addition, cancer-related exosomes modulate host immunity by changing the behavior of macrophages. This process promotes tumor progression and metastasis by releasing cytokines, inducing tissue remodeling, and promoting angiogenesis. Guo et al. (2019) reported that PCa-associated exosomes promoted tumor immune escape by impairing the cytotoxic function of lymphocytes and reducing the expression of NKG2D receptors in NK cells and CD8^+^ T cells (Fiaschi et al., 2012). Other studies have shown that tumor-derived exosomes activate Toll-like receptor 2 through membrane-connected heat shock proteins (HSPs) to increase the production of myelogenous suppressor cells interleukin-6, thus promoting autocrine phosphorylation of Stat3, enhancing immune system immunosuppression, and promoting PCa (Chalmin et al., 2010). The role of exosomes in the invasion and metastasis of PCa is shown in Figure 2.

The process of tumor invasion and metastasis is a dynamic and complex process that includes multiple simultaneous steps, or steps wherein one is evolved from other steps. With advances in the in-depth understanding of the mechanism of tumor invasion and metastasis, it was found that exosomes play an important role in the process of tumor invasion and metastasis, and the exosomes enrich many bioactive molecules, which can be used as biomarkers to monitor the invasion and metastasis of PCa.

## The Advantages of Liquid Biopsy for PCa Diagnosis

PCa is a slow-growing tumor with a high mortality rate in metastatic disease. In addition, 40% of patients diagnosed with PCa have no clinical symptoms. However, if PCa is diagnosed at an early stage, the 5-year survival rate is >99%, compared to if it is diagnosed in the late metastatic stage, wherein the 5-year survival rate is only 30%. Therefore, the early diagnosis of PCa can fundamentally improve patient prognosis.

Digital rectal examination (DRE), transrectal ultrasound, and serum PSA determination are the three basic methods for the clinical diagnosis of PCa. Among these, PSA is the gold standard for the clinical diagnosis of PCa. Serum PSA or closely related (−2) proPSA levels are widely used in the diagnosis of PCa (Sokoll et al., 2010). However, the PSA test is not accurate enough because the PSA level (≥4 ng/mL) of patients with BPH or prostate inflammation also increases. In men with PSA levels between 4 and 10 ng/mL, the specificity of this diagnostic method for PCa is only 20–40%. As a result, many patients have to undergo additional prostate biopsies. Prostate biopsy is an expensive and invasive procedure that causes intense discomfort to patients. In addition, over a quarter of men diagnosed with PCa have PSA levels within the normal range (≤4 mg/mL) (Heidenreich et al., 2013). Therefore, new non-invasive biomarkers are in great demand to replace this diagnostic method in clinical settings.

Liquid biopsy is less invasive than traditional surgical biopsy, detecting specific biomarkers in readily available samples of bodily fluids. Blood, urine, saliva, and other body fluids can be easily obtained during liquid biopsy. Therefore, liquid biopsy is considered a tool for identifying alternative biomarkers. Exosomes may be as a key biomarker in the early diagnosis and could be used for the personalized treatment and prognosis of patients with PCa (Vlaeminck-Guillem, 2018). Exosomes in the blood and urine contain specific components for PCa metastasis and progression. Exosomes are different from other circulation biomarkers because they originate from endosomes and are enriched in proteins, RNAs, and lipids. Exosome surface proteins can be used as antigenic determinants and can be recognized using different monoclonal or polyclonal antibodies. Currently, there are special detection methods to determine whether exosomes in plasma can be used as markers for PCa. In addition, several exosomal bioactive molecules have been clinically studied as potential cancer biomarkers (Lee et al., 2018). As unique biomarkers, several exosomal proteins in urine show high sensitivity and specificity in PCa, which may completely distinguish PCa patients from non-disease controls (McKiernan et al., 2018, 2016). Exosomes isolated from urine are a promising non-invasive biomarker source with potential application value in the diagnosis, prognosis, and monitoring of PCa.

**FIGURE 3:**
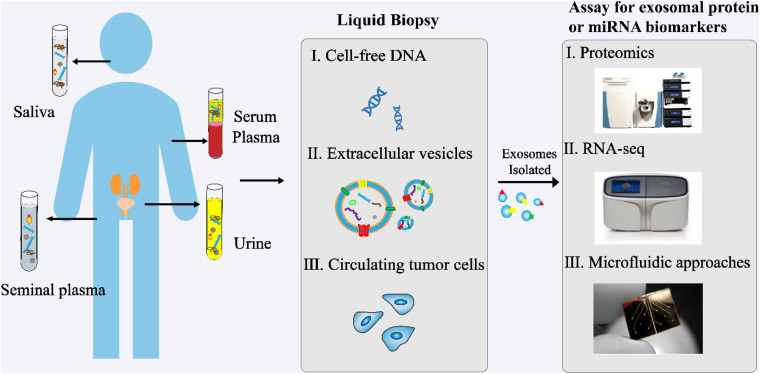
Analysis of exosomes in liquid biopsy of PCa. High concentrations of exosomes are found in various body fluids, including blood, urine, saliva, and seminal plasma. The proteins and RNAs enriched in exosomes reflect the specific physiological conditions and functions of their samples. These exosomes and their biomolecules are ideal biomarkers for liquid biopsy.

In recent years, exosomes have become a topic of great interest as a source of new biomarkers for liquid biopsies (Figure 3). Exosomes can overcome the limitations of previous biomarkers used for PCa. In addition, high concentrations of exosomes are found in various body fluids, including blood, urine, saliva, and seminal plasma. Proteins and RNAs enriched in exosomes can reflect the specific physiological conditions and functions of their samples. Therefore, exosomes and their biomolecules have good potential as ideal biomarkers for liquid biopsy.

## Exosome Bioactive Molecules as Biomarkers for PCa Detection

Recent studies have demonstrated that exosomes isolated from the TME are important factors that affect the progression of PCa (Saber et al., 2020). As carriers of proteins, RNAs, and lipids, PCa-derived exosomes have been reported to affect cancer cell proliferation, angiogenesis, survival, and immune escape (Vlaeminck-Guillem, 2018). Cancer cells communicate with each other or CAFs through substances carried by exosomes, which include signaling complexes, receptors, functional proteins, and genetic information that regulates signaling networks involved in cancer growth and invasiveness. In terms of recent studies on protein and miRNA biomarkers, this section focuses on exosomal proteins and miRNAs in PCa monitoring.

### Exosomal Protein Biomarkers for PCa Monitoring

A large amount of evidence indicates that proteins in exosomes play a crucial role in the invasion and metastasis of PCa (Table 1). In fact, exosome transfer proteins (e.g., Caveolin-1, PKM2, ITGA3, and ITGB1) from cells with strong invasive ability to weakly invasive cells have been found to increase PCa invasion and metastasis (Bijnsdorp et al., 2013; Hoshino et al., 2015; Dai et al., 2019; Lin et al., 2019).

**TABLE 1:** Exosome-associated proteins and their functional relevance in PCa invasion and metastasis.

**Protein**	**Source**	**Biological function**	**References**
Hypoxia-inducible factor 1α (HIF-1α)	Cell supernatant of PC-3 and LNCaP lines	Associated with PCa aggressiveness	Hasan et al., 2018; Wu et al., 2019
Integrin αvβ3 and Integrin αvβ6	Serum of PCa patients and cell supernatant of CWR22 and PC-3 lines	Pro-inflammatory effect on stromal cells	Fedele et al., 2015; Singh et al., 2016; Krishn et al., 2019
ITGA2	Cell supernatant of PC-3 and DU-145 lines	Change the expression of EMT-related factors	Gaballa et al., 2020
PKM2	Serum of PCa patients and cell supernatant of PC-3 and C4-2B lines	Educate bone stroma to promote bone metastasis	Dai et al., 2019
Hyal 1	Cell supernatant of 22RV1 line	Promote PCa progression and metastasis	McAtee et al., 2019
Caveolin-1	Cell supernatant of PC-3, DU-145, and 22RV1 lines	Change the expression of EMT-related factors	Lin et al., 2019
PLD2	Cell supernatant of PC-3 and C4-2B lines	Activate the proliferation and differentiation of osteoblasts	Lin et al., 2019
Integrin αvβ3 and Synaptophysin	Cell supernatant of PC-3 and LNCaP lines	Activate src phosphorylation and promote inflammation	Fedele et al., 2015; Hoshino et al., 2015; Singh et al., 2016
ITGA3 and ITGB1	Cell supernatant of PC-3 and LNCaP lines	Promote epithelial cell invasion and migration	Bijnsdorp et al., 2013; Hoshino et al., 2015
MMP-9 and MMP-14	Cell supernatant of PC-3 line	Promote PCa cell growth	Wang et al., 2009; Vlaeminck-Guillem, 2018
c-Src, IGF-1R, and FAK	Cell supernatant of PC-3, DU-145, and C4-2B lines	Promote PCa development and angiogenesis	Marx et al., 2001; Chang et al., 2007; DeRita et al., 2017
Rab1a, Rab1b, and Rab11a	Cell supernatant of C4-2B lines	Promote PCa cell growth	Abd Elmageed et al., 2014
Trop-2, vimentin, N-cadherin, and Integrin αvβ3	Cell supernatant of PC-3 line	Induce PCa cell invasion	Chowdhury et al., 2015; Fedele et al., 2015; Trerotola et al., 2015; El-Sayed et al., 2017; Krishn et al., 2019
CD63, CD81, HSP90, HSP70, TNF1α, IL-6, MMP2, MMP9, Annexin II, TSG101, Akt, ILK1, and β-catenin	Cell supernatant of PC-3 and LNCaP lines	Increase stemness, metastasis, and CAFs formation	Ramteke et al., 2015

Studies have shown that exosomes derived from LNCaP and DU-145 cells induce PCa cell proliferation, EMT, migration, and IL-8 secretion, and reduce the apoptosis of PCa cells (Waugh and Wilson, 2008; Hosseini-Beheshti et al., 2016; Souza et al., 2018). In addition, the information transmitted by exosomes derived from cancer cells in a hypoxic environment can directly participate in the invasion and movement of PCa cells in the latent period (Ramteke et al., 2015). Hasan et al. (2018) found that exosomal HIF-1α promotes the occurrence and progression of PCa metastasis by promoting the loss of E-cadherin (Wu et al., 2019). Exosomes released from different PCa cells confer different functions on recipient cells. Integrins on exosomes secreted by PC-3 (integrin αυβ6 and integrin αυβ3) and CWR22 (integrin αυβ3) cells were found to shuttle to DU-145 and C4-2B cells that do not secrete integrins, thus inducing their progression and invasion (Fedele et al., 2015; Singh et al., 2016). Gaballa et al. (2020) found that exosome-mediated ITGA2 promotes the migration and invasion of PCa cells by inducing EMT. Dai et al. (2019) found that exosome-mediated pyruvate kinase M2 (PKM2) is transferred from PCa cells to bone marrow stromal cells, and increases the secretion of CXCL12 in bone marrow stromal cells in a HIF-1α-dependent manner to promote the occurrence of a pre-metastatic niche, thus promoting the bone metastasis of PCa. McAtee et al. (2019) found that the exosomes of PCa containing hyaluronidase 1 (Hyal 1) stimulate the movement of prostate stromal cells through FAK-mediated integrin signaling. The overexpression of Hyal 1 allows PCa cells to speed up the transport rate of exosomes by changing cell surface integrins and cadherins, thereby enhancing their metastatic potential by increasing the mobility and proliferation of exosomes (McAtee et al., 2019). Lin et al. (2019) found that exosomal Caveolin-1 promotes the invasion and metastasis of PCa in an endocrine manner and induces the phenotype and EMT of cancer stem cells (CSCs) through the NF-κB signaling pathway. Borel et al. (2020) found that exosomal phospholipase D2 (PLD2) derived from the C4-2B cell line activates the proliferation and differentiation of osteoblast models by stimulating ERK1/2 phosphorylation and increasing the activity of non-specific alkaline phosphatase and the expression of osteoblast differentiation markers.

ITGA3, ITGB1, and ITGB4 are exosome proteins involved in the progression of PCa. These exosomes carry integrin α3 and integrin β1 to promote the migration and invasion of epithelial cells. In PCa cells, integrin αvβ3 is considered a biomarker of invasive PCa and is co-expressed with synaptophysin (Fedele et al., 2015; Hoshino et al., 2015; Singh et al., 2016). It has been reported that PCa-related exosomes deliver integrin αvβ3 to the TME, activate the phosphorylation of Src in recipient cells, and enhance the expression of S100 protein in stromal cells to promote inflammation, migration, and invasion (Bijnsdorp et al., 2013; Kawakami et al., 2015). It was found that PCa-derived exosomes do express MMP-9 and MMP-14 by stimulating ERK1/2 phosphorylation, inhibiting the apoptosis of PCa cells, enhancing their migration rate, and preparing for invasion and metastasis (Wang et al., 2009; Vlaeminck-Guillem, 2018). At the same time, there are other exosomal proteins, such as c-Src tyrosine kinase, IGF-1R, and FAK, which induce angiogenesis by stimulating VEGF transcription in the TME, playing an important role in PCa progression and angiogenesis (Marx et al., 2001; Chang et al., 2007; DeRita et al., 2017). Abd Elmageed et al. (2014) demonstrated that exosomes derived from PCa cells transport the RAS superfamily GTP enzymes Rab1a, Rab1b, and Rab11a into PCa patient adipose-derived stem cells (pASCs) in the TME, thus promoting the growth, cloning, and amplification of PCa cells. Fedele et al. (2015) found that exosomal proteins derived from PCa, such as Trop-2, vimentin, N-cadherin, and integrin αvβ3, induce the invasion of PCa cells (Chowdhury et al., 2015; Trerotola et al., 2015; El-Sayed et al., 2017). Ramteke et al. (2015) reported that proteins (CD63, CD81, HSP90, HSP70, annexin II, TGF-β2, TNF1-α, IL6, TSG101, AKT, ILK1, and β-catenin) secreted by PCa in anoxic environments promote fibroblast formation and cancer cell metastasis, indicating that improving our understanding of the changes in exosome production and the release mechanisms under hypoxic conditions, which would be helpful to prevent metastasis of PCa cells, may be important.

The above-mentioned exosomal proteins (Table 1) play an important role in cancer invasion and metastasis through different mechanisms of action. Therefore, researchers can choose to monitor the progression and metastasis of PCa by measuring changes in the expression of these exosomal proteins. In addition, novel proteins can be identified using modern proteomic technologies, adding value to the exosome biomarkers used for PCa monitoring.

### Exosomal miRNA Biomarkers for PCa Monitoring

Exosomal miRNAs are short-stranded non-coding RNAs with lengths of 17–25 nucleotides that are responsible for regulating gene expression. Evidence has shown that exosomal miRNAs regulate many biological functions in the TME, which are related to tumor progression, such as the tumor response of stromal cells and the growth, differentiation, proliferation, apoptosis, and invasion of tumor cells (Jiang et al., 2017).

Exosomes, as carriers of miRNAs, are also involved in the regulation of PCa progression. Wang et al. (2019) found that exosomal miR-26a derived from PCa cells significantly altered the expression of EMT-related factors and inhibited the metastasis and tumor growth of PCa. According to previous reports, the high expression of miR-141-3p and low expression of miR-125a-5p in plasma exosomes from PCa patients control the invasion and migration of PCa cells by regulating the activity of the PI3K/AKT/mTOR pathway. The ratio of miR-125a-5p/miR-141-3p is a good indicator of the activation of the PI3K/AKT/mTOR pathway, thus indirectly predicting the possibility of tumor occurrence and development (Li W. et al., 2020). It was found that exosomal miR-141-3p in PCa cells promoted the activity of osteoblasts, regulated the microenvironment of bone metastasis, and induced the bone metastasis of PCa (Ye et al., 2017). Li et al. (2019) found that exosomal miR-375 in LNCaP cells significantly promoted the activity of osteoblasts, thus promoting bone metastasis in PCa. Some studies have shown that exosomal miR-888 inhibits the expression of tumor suppressor genes RBL1, KLF5, Smad4, and TIMP2 in PCa cells in a 3′UTR-dependent manner, and promotes the growth and invasion of PCa cells (Hasegawa et al., 2018). Hashimoto et al. (2018) showed that exosomal hsa-miR-940 secreted by PCa induces bone marrow mesenchymal stem cells in the bone metastasis microenvironment to transform into an osteoblast phenotype through exosomes targeting ARHGAP1 and FAM134A, which promotes bone metastasis of PCa. Bhagirath et al. (2018) found that exosomal miR-1246 is a PCa tumor suppressor miRNA that is released via exosomes in the blood, inhibiting the activity of N-cadherin and vimentin, and thus inhibiting EMT. The overexpression of exosomal miR-1246 significantly inhibits the growth of transplanted tumors *in vivo*, increases apoptosis, and decreases the ability for proliferation, invasion, and migration (Bhagirath et al., 2018). Zhou et al. (2020) found that upregulating the level of miR-217 in exosomes secreted by PCa cells promotes cell proliferation and invasion, while upregulating the level of miR-23b-3p in exosomes inhibits cell proliferation and invasion, which plays a role by affecting the process of EMT, suggesting that they are potential therapeutic targets for the treatment of PCa.

PCa-derived exosomal miR-21-5p, miR-100-5p, and miR-139-5p have been reported to upregulate the expression of receptor activator for nuclear factor-κ B ligand (RANKL) and MMP receptor activator in fibroblasts and promote the growth and metastasis of PCa (Sanchez et al., 2016). Studies have also shown that exosomes secreted by PC-3 cells inhibit osteoclast differentiation by downregulating miR-214 and blocking the NF-κB signaling pathway. Therefore, increasing the level of miR-214 at the site of bone metastasis may reduce the aggressiveness of PCa (Duan et al., 2019). In addition, the expression levels of miR-21 and miR-141 in exosomes derived from patients with metastatic PCa are high, regulating osteoclast and osteoblast production and helping cancer cells overcome the low androgen condition of distant metastatic organs (Liu et al., 2016; Pan et al., 2017). Exosomal miR-125a induces the immune escape of tumor cells and promotes the growth and invasion of PCa cells by suppressing the proliferation of cultured macrophages in the TME (Kim et al., 2014). The AATF/Che-1 genome is similar to an epigenetic master switch that regulates cell cycle progression, checkpoint control, and apoptosis (Iezzi and Fanciulli, 2015). In addition, the AATF genome also contains regulatory non-coding miR-2909, which regulates key genes involved in host immunity, energy metabolism, and tumor progression (Malik et al., 2014; Kaul et al., 2015). Wani et al. (2017) have shown that the recruitment of miR-2909 in the urine exoskeletons of patients with PCa can be used as a non-invasive diagnostic marker for all characteristics of the severity of PCa. Exosome-associated miRNAs and their functional relevance in PCa invasion and metastasis are shown in Table 2.

**TABLE 2:** Exosome-associated miRNAs and their functional relevance in PCa invasion and metastasis.

**Nucleic acid**	**Source**	**Biological function**	**References**
miR-26a	Cell supernatant of LNCaP line	Change the expression of EMT-related factors	Wang et al., 2019
miR-125a-5p and miR-141-3p	Serum of PCa patients	Regulate the microenvironment of bone metastases and promote bone metastasis of PCa	Li W. et al., 2020
miR-375	Cell supernatant of LNCaP line	Activate the proliferation and differentiation of osteoblasts	Li et al., 2019
miR-888 Cluster	Cell supernatant of PC-3-ML line	Repress the tumor suppressor genes	Hasegawa et al., 2018
hsa-miR-940	Cell supernatant of C4-2B line	Induce extensive osteoblastic lesions in the bone metastatic microenvironment	Hashimoto et al., 2018
miR-1246	Cell supernatant of PC-3 line	Change the expression of EMT-related factors	Bhagirath et al., 2018
miR-217 and miR-23b-3p	Serum of PCa patients	Change the expression of EMT-related factors	Zhou et al., 2020
miR-21-5p, miR-100-5p, and miR-139-5p	Cell supernatant of primary cell cultures established from tissue of PCa patients	Induce fibroblast migration	Sanchez et al., 2016
miR-214	Cell supernatant of PC-3 line	Inhibit osteoclast differentiation and attenuates the invasion of PCa	Duan et al., 2019
miR-21 and miR-141	Serum of PCa patients and cell supernatant of LNCaP line	Affect osteoclastogenesis and osteoblastogenesis and help PCa cells to overcome androgen deprivation in long-distance metastasis	Liu et al., 2016; Pan et al., 2017
miR-125a	Cell supernatant of LNCaP line	Regulate tumor microenvironment	Kim et al., 2014
miR-2909	Urine of PCa patients	Regulates immunity, energy metabolism, and tumor progression	Wani et al., 2017

With the discovery of high concentrations of exosomal miRNAs, miRNAs have attracted attention as cancer biomarkers with potential applications in the diagnosis, prognosis, and monitoring of invasion and metastasis of PCa. The above-mentioned exosomal miRNAs are abnormally expressed during PCa invasion and metastasis, which creates favorable conditions for the invasion and metastasis of PCa. RNA sequence analysis was conducted to compare the differences in exosomal miRNAs from normal and metastatic cells. The results indicated that certain miRNAs should be applied as potential biomarkers in further experiments to obtain new ideas for the monitoring of PCa invasion.

### Exosomal lncRNA and circRNA Biomarkers for PCa Monitoring

Exosomes include proteins, miRNAs, lncRNAs, and circRNAs, which also play an important role in the occurrence and development of PCa. Although there are few studies on the lncRNAs and circRNAs of PCa exosomes, it has been pointed out that they may become promising biomarkers for tumor diagnosis and monitoring metastasis. For example, Li et al., found that circ_0044516 was upregulated in PCa blood exosomes and PCa cell exosomes. Although inhibiting the expression of circ_0044516 in PCa cells reduced their growth and metastasis, the mechanism underlying this action remains unclear (Li T. et al., 2020). Although studies on exosomal lncRNAs in PCa are scarce, a study by Wu et al. (2020) provided convincing evidence about exosomal lncRNAs as potential diagnostic markers using meta-analysis. Wang et al. (2018) proved for the first time that the detection of tumor-derived exosomal lncRNAs in plasma led to the identification of biomarkers with potential diagnostic applications in PCa, among which SAP30L-AS1 and SChLAP1 are promising markers and show potential for the detection and stratification of PCa. As special molecules, lncRNA and circRNA in exosomes could be promising bioindicators for the diagnosis and monitoring of PCa metastasis.

## Current Challenges for Liquid Biopsy and Exosome Research

Liquid biopsy is a simple, convenient, and non-invasive or minimally invasive detection method with high detection sensitivity and specificity. However, in the process of collecting samples from liquid biopsies, the sample quality may be unqualified due to the improper selection of test tubes, the improper storage of samples, and untimely treatment, which may lead to false-negative test results. Liquid biopsies have a low concentration of samples detected in the early stages of cancer, which requires a detection technique with an extremely high sensitivity. Liquid biopsy depends on cells, exosomes, nucleic acids, proteins, and other substances released or exfoliated from tumor tissue, which are detected in body fluids outside the tissue. The low abundance of the sample is inherent. However, the low sensitivity caused by the low abundance of samples is the biggest bottleneck in the clinical application of liquid biopsies, with false-negative cases occurring often in clinical settings. Although enrichment, amplification, and other methods can improve the sensitivity of detection, such as ddPCR and next-generation sequencing (NGS), this may also lead to incomplete information, gene mismatch, and false positives. Therefore, the standardized operation of sampling, transportation, extraction, on-board, data analysis, quality control of key nodes, the development of new technologies for liquid biopsy are the top priorities for the study of liquid biopsies in clinical settings. Testing firms do not implement a unified liquid biopsy standard; therefore, even for the same disease detection, the results may vary according to the choice of gene locus design, gene sequencing depth, and bioinformatics analysis code.

Exosomes have been extensively studied as biomarkers for liquid biopsy. However, in the study of exosomes, a very challenging problem is the lack of standardized separation and purification methods for exosomes that cannot effectively and selectively separate exosomes (Yang et al., 2019). Current research methods struggle to effectively conduct qualitative and quantitative analyses of exosomes, significantly affecting the follow-up experimental results, which results in a poor repeatability and reproducibility of the data (Gheinani et al., 2018). Therefore, the purification methods of exosomes need to be optimized and standardized, and there is a general consensus among exosome researchers to solve this problem urgently.

Since both tumor cells and normal cells produce exosomes, the isolation of exosomes from tumor cells remains a considerable challenge. Exosome markers, such as CD9, CD63, CD81, and TSG101, are commonly used to verify isolated exosomes. However, these markers are not suitable for identifying exosomes from specific disease sources. As such, it is necessary to identify markers to isolate cancer-specific exosomes to improve the purity of exosomes.

## Conclusion and Future Perspectives

Advances in science and technology have opened new paths for understanding the mechanisms underlying the occurrence, development, and metastasis of cancer, as well as providing novel treatment options to improve the survival rate of cancer patients. An important scientific direction in recent years has involved the elucidation of the various roles of exosomes in cancer biology and their clinical applications.

A large number of studies have shown that proteins, RNAs, and lipids in exosomes are highly involved in communication between cells. Exosomes promote cancer progression by transporting different types of proteins or RNAs to their target cells through different mechanisms. Thus, the study of the biologically active molecules of exosomes secreted by PCa patients’ body fluids and/or PCa cells is likely to aid in the development of novel strategies for the monitoring of cancer invasion and metastasis.

The study of exosomal miRNAs and proteins in PCa liquid biopsy is currently at an early stage. In addition, the complicated nature of the process of sampling, isolating, transporting, and storage of exosomes in body fluids is likely affecting subsequent experimental analysis results. For example, storage and retrieval conditions of body fluids and isolated exosomes can affect the characteristics of exosomes, including stability, number of particles, aggregation and function (Yuana et al., 2011, 2015; Vila-Liante et al., 2016). A combination of a lipophilic cationic dye (LCD) probing EVs and polychromatic flow cytometry (PFC) can identify, enumerate and separate EVs from different cells origins and fresh peripheral blood samples (Marchisio et al., 2020). Currently, the standardized separation method of exosomes in body fluids is not uniform. Therefore, there is an urgent need to establish standardized separation and purification methods for the isolation and analysis of exosomes. At the same time, an accurate analysis platform is needed to perform subsequent miRNA and protein analyses. With the discovery of new biomarkers, new platforms are being developed for the detection of exosome biomarkers, such as microfluidics. However, there are no uniform standards for the use of specific technologies, standardized methods, sample collection, and processing. This makes the results of preclinical research and clinical research inconsistent, resulting in many new biomarkers not being applied in clinical settings. To ensure that new biomarkers are tested in clinical trials, these obstacles need to be overcome with the development of new technologies.

Despite many challenges, exosomal miRNAs and proteins are promising biomarkers for monitoring the invasion and metastasis of PCa. However, there is a need to develop clinical usage equipment and techniques to detect small volumes of samples, such as ZnO chip biosensor. The value of this microfluidic colorimetric tool is that the detection results can be viewed with a smartphone or computer, which eliminates the requirement of extensive lab equipment for exosome screening (Chen et al., 2018). The application of new technologies such as microfluidics will be used for translational research in future research, which will allow research findings to transition from the bench to clinical settings.

## Author Contributions

ZG, TF, and YL conceived the manuscript. ZG wrote the manuscript, drew the figures and tables, and was a major contributor in writing. BP, JL, NG, TF, and YL revised the manuscript. All authors read and approved the final manuscript.

## Conflict of Interest

The authors declare that the research was conducted in the absence of any commercial or financial relationships that could be construed as a potential conflict of interest.
